# A Systematic Survey of Data Augmentation of ECG Signals for AI Applications

**DOI:** 10.3390/s23115237

**Published:** 2023-05-31

**Authors:** Md Moklesur Rahman, Massimo Walter Rivolta, Fabio Badilini, Roberto Sassi

**Affiliations:** 1 Dipartimento di Informatica, Università degli Studi di Milano, 20133 Milan, Italy; 2 School of Nursing, University of California, San Francisco, CA 94143, USA; 3 AMPS-LLC, New York, NY 10025, USA

**Keywords:** ECG augmentation, artificial intelligence, electrocardiogram, AI in cardiology, data augmentation

## Abstract

AI techniques have recently been put under the spotlight for analyzing electrocardiograms (ECGs). However, the performance of AI-based models relies on the accumulation of large-scale labeled datasets, which is challenging. To increase the performance of AI-based models, data augmentation (DA) strategies have been developed recently. The study presented a comprehensive systematic literature review of DA for ECG signals. We conducted a systematic search and categorized the selected documents by AI application, number of leads involved, DA method, classifier, performance improvements after DA, and datasets employed. With such information, this study provided a better understanding of the potential of ECG augmentation in enhancing the performance of AI-based ECG applications. This study adhered to the rigorous PRISMA guidelines for systematic reviews. To ensure comprehensive coverage, publications between 2013 and 2023 were searched across multiple databases, including IEEE Explore, PubMed, and Web of Science. The records were meticulously reviewed to determine their relevance to the study’s objective, and those that met the inclusion criteria were selected for further analysis. Consequently, 119 papers were deemed relevant for further review. Overall, this study shed light on the potential of DA to advance the field of ECG diagnosis and monitoring.

## 1. Introduction

Cardiovascular diseases (CVDs) are significant contributors to worldwide fatalities, accounting for approximately 33% of all deaths across the world [1]. Because of its low cost, simplicity, and non-invasive nature, the electrocardiogram (ECG) is the most used technique for diagnosing and monitoring CVDs in both clinical and telemedicine settings [2]. The ECG provides specific information about the structure and electrical activity of the heart by detecting alterations in the shape and timing of the captured waveforms. In clinical applications, prompt and precise ECG interpretation is essential. For instance, in patients suffering from myocardial infarction (MI), the quicker the abnormal condition is detected, the higher the chance of avoiding threats to life and recovery. However, due to the complexity of the ECG signal and the overlapping noise, diagnosing certain conditions may take a long time (for instance, for rare events where 24 h or longer Holter recordings are necessary) and require significant human review [3]. Moreover, due to the unique individual characteristics of the ECG, features may vary significantly in different patients under different physiological conditions, posing considerable difficulties for the recognition of ECG patterns. Signal interpretation can thus be a time-consuming and complex process which leads to subjective ambiguity and human mistakes in their analysis, even for highly-trained specialists. To tackle these problems, automatic systems for interpreting ECG signals have been developed since the 1960s [4].

The accurate and automatic detection of CVDs from ECG signals is a topic of relevant clinical interest, particularly with the increasing spread of new wearable technology, the modern implementation of artificial intelligence (AI), and the growth of digital health solutions. Computer-assisted analysis of ECG signals allows achieving objective results with less room for inter-operator and operator-specific errors. The three main components of an automated system for ECG interpretation are signal preprocessing, feature extraction, and classification. In the preprocessing phase, signals are denoised, segmented, and normalized. In the feature extraction phase, time domain and frequency domain features are extracted. Lastly, the extracted features are used to classify the signals into distinct diseases.

In recent times, AI, but especially deep learning (DL), has shown remarkable results in the classification of ECG signals [5,6]. This is likely due to the robust capability of DL for feature extraction. Deep neural networks consist of a composition of layers (i.e., mathematical functions), which makes DL highly adept at processing complex biomedical signals, including ECG signals. The composition, indeed, resembles the mathematical steps typically employed in traditional ECG signal processing algorithms. For example, the famous Pan–Tompkins algorithm [7], which detects the timing of each heartbeat within an ECG signal, involves the use of a first filter, a rectification operation, and a second filter: these steps are exactly what is commonly employed in DL architectures as a sequence of convolutional layers and non-linear activation functions. Moreover, with each layer, more abstract and high-level features can be extracted which can be leveraged to identify CVDs. As a result, compared with conventional machine learning (ML), and provided sufficient expression capability, DL potentially reaches a better ability to represent the most relevant features of complex data.

DL models are data-hungry by nature, i.e., the performance of a DL model relies heavily on the availability of high-quality labeled datasets and a large number of training samples. Training a DL model with insufficient data or an unbalanced dataset may result in poor performance, a non-converging training phase, and biased classification outcomes. To circumvent these issues, the DL model needs a large and balanced dataset. However, obtaining a large number of training samples and a balanced dataset is difficult due to, for example, the low incidence rate of abnormal cardiac events and the availability of expert cardiologists to accurately label (annotate) the waveforms. In addition, the number of ECG recordings annotated by cardiologists is limited because only expert physicians are capable of accurately annotating the recordings. To guarantee performance, the building of a DL model is frequently accompanied by a data augmentation (DA) technique, which provides additional and presumably non-redundant training data by deforming the training set. DA aims to improve the generalization ability of trained models by reducing overfitting and broadening the decision boundary of the model.

In image recognition, DA has already reached a stable state. To improve the performance, most state-of-the-art convolutional neural network (CNN) architectures use some form of DA technique. For example, residual networks (ResNet) use color augmentation, scaling, and cropping [8], DenseNet uses mirroring and translation [9], and inception networks use mirroring and cropping [10]. Unfortunately, in the case of ECG data, such kinds of random transformations are not effective because the relative amplitudes of different cardiac beat segments (P waves, QRS complex, T waves, ST segments etc.) carry relevant information for the diagnosis. For example, time inversion assumes that it is normal that the sequence of ECG waves is reversed. Although these can be effective for augmenting the data (e.g., spectral), these techniques are likely not effective for ECGs. Another example could be randomly cropping and merging the ECG data which may easily transform the normal (sinus) rhythm into an arrhythmic pattern.

**Figure 1:**
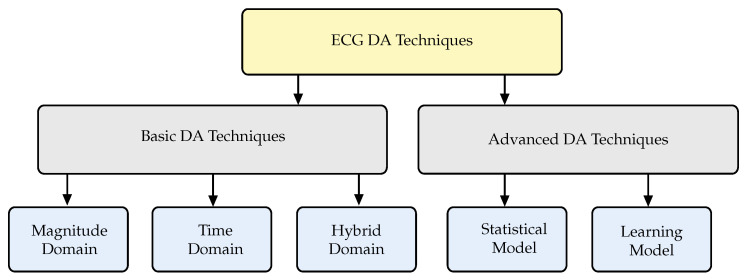
Taxonomy of ECG DA techniques.

To the best of our knowledge, no comprehensive review of DA techniques of AI applications on ECG signals has been carried out yet. Since its practical significance and potential for the development of ECG classification models, we considered the DA techniques of the AI methods applied in ECG classification worth a review. In this study, papers were systematically reviewed and their characteristics were highlighted. By reviewing all methods, a taxonomy of the ECG DA methods is proposed for the first time and illustrated in Figure 1. The taxonomy mainly breaks down into two categories: basic DA techniques and advanced DA techniques. The basic DA techniques include all methods based on random and non-random transformations of the ECG signal in the magnitude domain, time domain, and time–magnitude domain. Conversely, advanced DA techniques include alternative methods to model the data such as, for example, statistical models and learning-based models. Advanced DA techniques follow the distributions of features present in the dataset to produce novel pattern combinations. Many statistical models, such as the Gaussian mixture model (GMM) and Markov chain (MC) models have been proposed for generating new samples [11,12,13]. Learning-based models such as variational autoencoder (VAE) [14] and generative adversarial networks (GANs) [15], are relatively new models developed recently for the generation of ECGs. The paper is structured as follows:

- A comprehensive review of the latest techniques for analyzing ECG signals using DA methods.
- A detailed taxonomy and categorization of ECG DA techniques, along with their various applications, datasets, and AI techniques.
- A comprehensive discussion of research gaps and open issues in the field that need further investigation.

## 2. Method

### 2.1. Literature Search Strategy

A comprehensive search of the published research was conducted through three databases: IEEE Xplore, PubMed, and Web of Science. The search criteria covered a broad spectrum of aspects, including signal types (i.e., ECG), various AI techniques, and diverse DA techniques. To ensure the relevance and accuracy of the findings, only articles published in the last ten years (between 1 January 2013 and 31 January 2023) in peer-reviewed English language journals, conference proceedings, chapters, and magazine articles were included. The comprehensive search query, including the specific search terms used, is detailed in Table 1.

### 2.2. Study Selection

**Figure 2:**
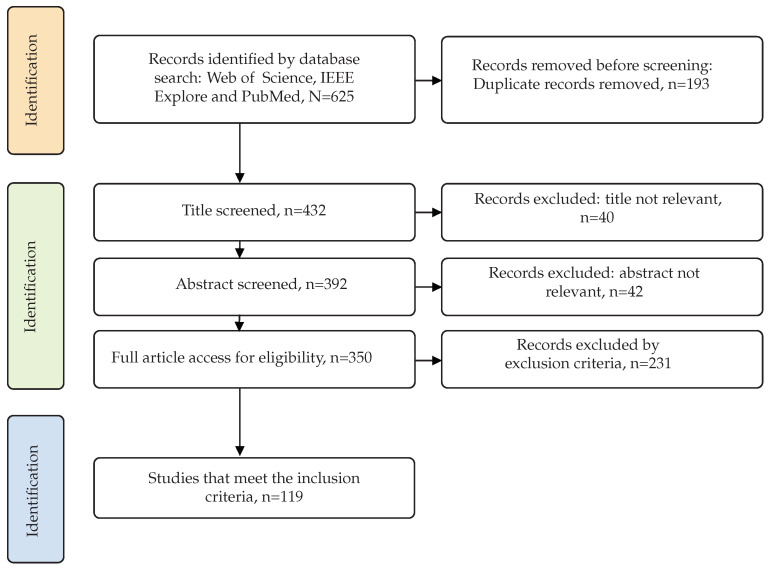
The search method for identifying relevant studies.

In order to ensure a rigorous and systematic approach to select articles, we followed the guidelines set forth by the Preferred Reporting Items for Systematic Reviews and Meta-Analyses (PRISMA) [16]. First, we utilized reference manager software to eliminate the duplicates. We then thoroughly screened the remaining works by evaluating titles and abstracts, and subsequently conducted a full reading of the selected papers to apply inclusion/exclusion criteria. To provide transparency and clarity of the selection process, a flowchart summarizing our research is reported in Figure 2. This method enabled us to effectively narrow down our options and identify studies that were directly relevant to our research objectives.

### 2.3. Results of the Research

Implementing the search query and inclusion/exclusion criteria, we initially obtained a list with 625 articles; we then excluded 193 duplicates using reference manager software or manual review. After screening the remaining 432 papers based on their titles and abstracts, 350 papers were selected for full-text evaluation according to our inclusion/exclusion criteria. The inclusion and exclusion criteria are presented in Table 2. Ultimately, 119 papers met the criteria and were kept for further analysis.

In the following sections, we present the major outputs of the literature review.

## 3. ECG Applications and Datasets

### 3.1. Typical ECG Applications

The most common implementation of AI in ECG analysis is automatic ECG interpretation [14,15,17,18]. Other important applications include localizing and annotating specific rhythms and beats, which can aid in the detection of conditions such as MI [13] and fetal heart rate series classification [19]. Moreover, recent advancements in biometric-based human identification show great promise for accurate recognition based on ECG data [20,21]. In addition, ECG analysis can also be utilized for detecting emotions and stress [22], pain [23], sleep-apnea [24,25], identification of COVID-19 infections [26,27,28,29], assessment of signal quality [30,31], and many other potential applications. In this study, we considered all applications as long as they investigated on DA of ECG via AI techniques.

### 3.2. Datasets

Most of the reviewed studies used a few ECG databases. The MIT-BIH AD database was used in 46% of the studies, followed by Physionet-2017 at 13%, PTB at 7%, Physionet-2020 at 5%, and Physionet-2021 at 3%. INCART, CPSC-2018, and PTB-XL were used in 2% of the studies each. The specific characteristics of these databases are hereafter described in detail.

- MIT-BIH AD: The MIT-BIH Arrhythmia Database contains a collection of 48 ECG ambulatory records of two leads, each spanning 30 minutes, gathered during the period from 1975 to 1979 [32]. These recordings, sampled at 360 Hz with 11-bit resolution over a 10 mV range, were collected from 47 individuals who were subjected to testing in the BIH Arrhythmia Laboratory. Within this dataset, we find several different types of cardiac abnormalities (CA), including but not limited to atrial fibrillation (AF), atrial bigeminy, atrial flutter, ventricular premature beat, right bundle branch block (RBBB), and left bundle branch block (LBBB).
- PhysioNet-2017: This dataset is a comprehensive collection of 8528 single-lead ECG data records obtained from 3658 individuals [33]. The ECG data are uniformly sampled at a rate of 300 Hz and span a duration of 9 to 61 seconds. The dataset encompasses four distinct rhythm categories, namely normal, AF, noise, and other.
- INCART: The St. Petersburg INCART dataset consists of 75 records extracted from 32 24-h Holter recordings where patients were diagnosed with various heart complications such as coronary artery disease, ischemia, conduction abnormalities, and arrhythmia. The records are sampled at a frequency of 257 Hz, ensuring that subtle changes in heart function are captured. Each record spans 30 min and contains 12 standard leads.
- CPSC-2018: The China Physiological Signal Challenge-2018 dataset is a comprehensive collection of 6,877 recordings of 12-lead ECG data, encompassing a diverse range of patients across genders and medical conditions [34]. The recordings were gathered from 11 hospitals, contributing to data’s diversity. Each ECG recording is sampled at 500 Hz, providing high-resolution physiological signal data for analysis. The recordings range in length from 6 to 60 s. This dataset comprises of nine different types of CAs, including AF, LBBB, RBBB, normal, premature atrial contraction, premature ventricular contraction, intrinsic paroxysmal atrioventricular block, ST-segment depression, and ST-segment elevation.
- PTB: The PTB dataset comprises 549 ECG records consisting of 15 leads (12 standard leads and 3 Frank leads) obtained from 290 individuals [35]. The records were sampled at a rate of 1000 Hz with 16-bit resolution. Each individual has up to five records, which allows for a longitudinal view of their health status. Among the subjects, 216 have been diagnosed with one of 8 different types of heart diseases, which include MI, cardiomyopathy/heart failure, bundle branch block, dysrhythmia, myocardial hypertrophy, valvular heart disease, and myocarditis. The remaining 52 individuals represent a healthy control group, which serves as a point of reference for comparison. However, the health status of 22 individuals remains unknown.
- PTB-XL: The PTB-XL dataset, a comprehensive collection of clinical ECGs, comprises 21,837 records taken from 18,885 patients [36]. These ECGs are 10 s in length and were captured at two different sampling rates, 100 Hz and 500 Hz, with 16-bit resolution, ensuring that the data were of high accuracy. Within this dataset, there are several distinct ECG rhythms and abnormalities, including normal, MI, conduction disturbance, and hypertrophy.
- PhysioNet-2021: The Physionet-2021 includes 12-lead ECG recordings from a large cohort of 6877 patients suffering from various CAs [37]. These recordings have been collected from six different hospital systems, located in four different countries spread across three continents. The dataset is available publicly as training data, with over 88,000 ECGs shared for this purpose. Some of the previously described databases were later included and are now part of Physionet-2021 (e.g., INCART, PTB, and PTB-XL).

## 4. Basic Data Augmentation Methods

The concept of basic DA techniques of ECGs originally comes from the random transformation of image and time series, such as scaling, flipping, noise addition, etc. There are three basic DA techniques for ECGs: time domain, magnitude domain, and hybrid domain. Time domain transformations change the ECG along the time axis, i.e., the data points on the ECG are moved to different time steps than the original sequence. Magnitude domain transformation is different from time domain transformation because the time steps stay the same while only the values of the elements (mV) change. For example, scaling, adding noise, dropping, etc., are all ways to change the values of the elements. Hybrid methods use both time and magnitude domains. Namely, basic DA generates pattern $x^{{}^{\prime }}$ using some random transformation functions.

(1) $$x^{{}^{\prime }}\leftarrow f\left(x\right)$$

where x is defined as $x={\left[x_{1},x_{2},\cdots ,x_{N}\right]}^{⊺}$ with *N* the number of time steps from the original dataset. Each $x_{n}$ represents the ECG amplitude at time *n* for each of the *L* measurements collected by multiple electrodes concurrently. For example, the standard clinical ECG is typically stored in a N×12 matrix (or its transpose). Based on the search query, we obtained several papers that are related to the basic DA method. The summary of basic DA is tabulated in Table 3. In the following, we provide details about the most commonly used basic DA operations in the paper we analyzed.

- Noise addition: The ECG signal x is modified by adding Gaussian random noise n. The noise n is generated by a random generator with a mean of 0 and a standard deviation of σ. Mathematically, the generated signal can be expressed as: x+n [19,38,39,40,41,42,43,44,45].
- Scaling: Each lead of the ECG signal is scaled by a random factor that is drawn from a normal distribution [21,25,38,39,40,42,46]. The operation is typically performed by multiplying the ECG signal by a diagonal matrix.
- Time inversion: Given the ECG signal x, the temporally inverted version of the signal is expressed as $x^{{}^{\prime }}={\left[x_{N},x_{N-1},\cdots ,x_{1}\right]}^{⊺}$ [38,40,41,47].
- Spatial inversion: The amplitude of the ECG x is multiplied by −1, causing a spatial inversion of the ECG. The transformed signal can be mathematically expressed as −x [38,40,41].
- Time-spatial inversion: Temporal–spatial inversion is a fusion of spatial and temporal inversion. Temporal–spatial reversal performs a horizontal reverse on the segments that have been vertically reversed [41,47,48].
- Permutation: The ECG signal is split into multiple segments and shuffled so as to randomly alter the temporal position of each segment and then recombined [38,40].
- Dropping: Masking the input signal randomly with a certain probability [38,40,44,49].
- Cutout: Randomly cut out (set to zero) a portion of the input signal with the width of magnitude × signal length [19,38,40,50] (same as dropping, but with each portion having the same given length).
- Sine: A sine wave is added to the ECG signal. The sine wave’s frequency and amplitude are chosen randomly [38,40].
- Square pulse: The ECG signal is added with a square pulse whose frequency and amplitude vary randomly [38,40].
- Time warping: Randomly chosen segments of the original ECG signals are stretched or compressed along the time axis. Random signal segments along the time axis are stretched and compressed by dynamic time warping [38,51].
- Baseline wandering: To create baseline wandering, different sinusoidal signals with random low frequencies and phases are generated and added to x [51,52].
- Lead removal: Lead removal is the process of picking a single lead at random and setting all of its time signal values to zero [52] (similar to dropping and cutout, but setting to zero an entire lead at once).
- **Lead order shuffling**: Lead order shuffling involves changing the placement order of all leads, or a subset, in a random fashion [39,52].
- High-pass filter: High-pass filtering employs a Butterworth filter with a fixed cutoff frequency (e.g., 0.5 Hz) to filter signals and eliminate baseline wander noise across all leads [52].
- Low-pass filter: A low-pass filter, specifically a Butterworth filter with a certain cutoff frequency (e.g., 47 Hz), is used to eliminate high-frequency noise from the noise for all leads [52]. Sometimes, this operation is referred to as Gaussian blur, as a one-dimensional Gaussian kernel is employed to “blur” (low-pass filter) the signal for all leads.
- Band-pass filter: A band-pass filter eliminates baseline drift and high-frequency signal components for all leads by employing a Butterworth filter with a certain low cut-off frequency, e.g., 0.5 Hz and a high cut-off frequency e.g., 47 Hz [39,45,52].
- Sigmoid compression: Sigmoid compression applies a sigmoidal activation function to the ECG signal for all leads [52].
- Powerline noise: Powerline noise refers to the interference pickup from powerlines at f=50 Hz (or 60 Hz) and its higher harmonics. While powerline noise can be a nuisance for ECG, it can be added to the original signal as a form of DA to increase the variability of ECG signal [46,51,53].
- Electromyographic (EMG) noise: EMG noise indicates the high-frequency noise induced by muscle contractions. Simulated EMG noise is added to the clean ECG signal using an appropriate signal processing technique, such as adding the two signals together or convolving the ECG signal with the EMG noise signal [51,53].
- Baseline shift: Baseline shift refers to changes in the baseline that occur as a result of variations in electrode–skin impedance brought by electrode movements. In this operation, a direct current offset can be added to the ECG signal to simulate baseline shift noise. The direct current offset is randomly generated and varies within a certain range to make it more realistic [46,51,53].
- Peak alteration: Peak alteration in ECG refers to any change or deviation from the normal shape and duration of the QRS complex or T-wave peaks in an ECG [21,54].
- Mix-up: New signals are generated by linearly interpolating two other real signals, using different weights for each one [55].

**Table 3:** Summary of basic DA methods for ECG classification using AI techniques.

Type	Lead	Input	Classifier	Improvem. after DA	Dataset	Refs.
CA	12	ECG	CNN	2.24%	Physionet-2020	[56]
CA	12	ECG	CNN-LSTM	3%	Physionet-2020	[39]
CA	12	ECG	ResNet	−0.063–2.54%	CPSC-2018	[52]
CA	12	ECG	CNN	–	Physionet-2020	[50]
CA	12	ECG	CNN	–	Physionet-2020	[57]
CA	12	ECG	ResNet	1.4–3.5%	ICBEB and PTB-XL	[46]
CA	1	ECG	CNN	–	MIT-BIH AD	[58]
CA	1	Spectral	Residual Attention	0.8%	MIT-BIH AD	[59]
CA	12	ECG	CNN	7.73%	Physionet-2021	[40]
CA	1	ECG	CNN	–	MIT-BIH AD	[60]
CA	12	ECG	ResNet	40%	INCART	[54]
CA	2	ECG	CNN	2.3%	Physionet-2017	[61]
CA	1	Spectral	CNN	–	MIT-BIH AD	[62]
CA	1	ECG	CNN	0.028%	MIT-BIH AD	[63]
CA	1	Spectral	CNN	–	MIT-BIH AD	[64]
CA	12	ECG	CNN	–	Physionet-2020	[65]
CA	8	ECG	CNN	–	Private	[43]
CA	12	ECG	CNN	1%	Physionet-2020	[66]
CA	12	Spectral	CNN	4.64%	PTB	[67]
CA	1	Spectral	CNN	–	Physionet-2017	[68]
CA	1	ECG	CNN	5%	MIT-BIH AD	[47]
CA	12	ECG	CNN	–	Physionet-2021	[45]
CA	1	ECG	BeatGAN	0.28%	MIT-BIH AD	[69]
CA	1	ECG	ResNet-LSTM	–	MIT-BIH AD, AFDB and Physionet-2017	[70]
CA	1	Spectral	Residual-Attention	–	MIT-BIH AD and Supraventricular Arrhythmia	[71]
CA	1	Spectral	CNN	–	MIT-BIH AD	[72]
CA	1	ECG	LSTM	42%	Physionet-2017	[73]
CA	2	ECG	CNN-RNN	–	Private	[74]
CA	1	ECG	CNN-LSTM	3%	MIT-BIH AD	[75]
CA	1	ECG	CNN-RNN	1.91%	Physionet-2017	[55]
CA	–	Spectral	CNN	–	MIT-BIH AD and PTB	[76]
CA	1	ECG	CNN	–	Physionet-2017	[44]
CA	1	ECG	CNN	–	Physionet-2017	[77]
CA	1	ECG	CNN	–	Physionet-2017	[49]
CA	1	ECG	ResNet-RNN	–	Physionet-2017	[78]
CA	12	ECG	CNN	–	Physionet-2021	[79]
CA	1	ECG	CNN	0.62–5.61%	MIT-BIH AD	[80]
CA	1	Spectral	Transformer	–	MIT-BIH AD	[81]
Biometric	1	ECG	CNN	–	CYBHi and UofTDB	[82]
Biometric	1	ECG	CNN	0.19%	PTB and LivDet2015 [83]	[84]
Biometric	1	ECG	CNN	12%	Physionet-2018	[21]
Frailty Identification	1	ECG	LSTM	3.2%	Private	[42]
Sleep apnea	1	ECG	CNN	–	Private	[25]
Peak detection	2	ECG	CNN	2.5%	MIT-BIH-NST	[85]
QA	1	ECG	CNN	2%	Physionet-2017	[86]
QA	12	Spectral	CNN	2.91%	PhysioNet-2011	[30]
QA	2	ECG	U-Net	–	QT [87]	[31]
Cardiac auscultation	2	Spectral	CNN	2–9%	Private	[88]
COVID-19	12	Image	CNN	–	COVID-ECG [89]	[27]
COVID-19	12	Image	CNN	–	COVID-ECG [89]	[29]
COVID-19	12	Image	CNN	−0.02%	COVID-ECG [89]	[28]
Emotion	1	ECG	CNN-SVM	20%	MAHNOB-HCI [90]	[41]
Emotion	1	ECG	CNN	59%	Dreamer	[91]
Fetal ECG	1	ECG	LSTM	10%	NIFECGC	[19]

## 5. Advanced Data Augmentation Techniques

Basic DA often alters the properties of ECG signals, resulting in the creation of “noise” rather than augmenting the dataset with meaningful samples. These augmented samples may have detrimental effects on ECG classification. For example, in [52], the authors reported that horizontal flipping and vertical flipping DA operations have detrimental effects on their classifier. To tackle the limitations of basic DA techniques, advanced DA techniques can be a reasonable alternative. Based on the search query, we obtained several papers that are related to advanced DA methods. The summary of advanced DA is tabulated in Table 4. Advanced DA techniques can be categorized into two types: statistical generative models and learning-based models. These two approaches are described in the following subsections.

### 5.1. Statistical Generative Model

ECG DA approaches based on statistical generative models typically involve modeling the dynamics of the ECG with, in fact, statistical models. For example, in [11], the authors proposed a GMM to solve the class imbalance issues of the AF detector. The GMM model showed better performance compared to oversampling the minority class. Silva et al. [12] designed a cardiorespiratory signal synthesizer by conditional sampling from a multimodally trained stochastic system of Gaussian copulas integrated with an MC. Zhu et al. [13] proposed a novel DA technique that took into account both probability distribution and geometry. In their technique, they introduced variations to the data distribution along the geodesic in a Wasserstein space, which is a mathematical concept used to measure the distance between two probability distributions. To calculate the ground metric of the Wasserstein space, they analyzed the cardiovascular characteristics of ECG signals, enabling them to compare their geometry. Then, the augmented samples were fed to the multi-feature transformer mode with real samples. The result was a significant improvement in performance: the AUCROC on the PTB-XL dataset increased by 6–17% compared to the unaugmented dataset.

### 5.2. Learning Based-Models

In the field of AI, DL-based generative models have emerged as a powerful tool for generating diverse data samples. These models have received widespread attention due to their ability to produce high-quality synthetic data that resemble real-world data. While there are several generative models available, not all of them have been utilized for ECG DA. Therefore, in the following subsection, we focused on the specific methods that have been employed for DA, which can potentially help to overcome the challenges of limited labeled ECG data in various AI-based ECG applications.

#### 5.2.1. Embedding Space

ECG DA techniques should not only be able to make diverse samples but also be able to imitate the features of real ECG. Due to the manifold unfolding in feature space, it is hypothesized that simple transformations applied to encoded inputs instead of the raw inputs would yield more convincing synthetic data. For example, Zhang et al. [48] used basic DA techniques for representational learning in the embedding space. Their learning model comprises two modules: an encoder and a classifier. The encoder generates representations using the temporal–spatial reverse detection approach, while the classifier is responsible for performing the temporal–spatial reverse detection task during the learning phase. After completing the learning process, the trained encoder is transferred to the second stage (the classifier) to be applied in different downstream tasks.

#### 5.2.2. Deep Generative Models

To generate realistic high-dimensional data, such as images, time series, and sequence data, deep generative models (DGMs) have lately demonstrated promising results. Based on the obtained results regarding the ECG, we can categorize DGMs into two broad categories: encoder–decoder networks and generative adversarial networks. In the following sections, we provide details about these two DGMs.

Variational Autoencoder (VAE): The VAE is a powerful DL architecture that has revolutionized the field of unsupervised learning. At the heart of a VAE, there are three critical components: an encoder, a decoder, and a loss function. The encoder and decoder are two distinct types of neural network, each responsible for encoding high-dimensional or structural inputs into a lower-dimensional latent space and decoding them back into high-dimensional outputs, respectively. The loss function used in VAEs is the negative log-likelihood, augmented with a regularizer to ensure that the generated outputs remain consistent with the input data. By sampling vectors from the latent space and transforming them through the decoder, VAEs can generate entirely new patterns, making them a powerful tool for data synthesis and augmentation. In [14], the author used vector quantized VAE (VQ-VAE) to augment the training samples of spectral images of 12 leads ECG. They reported that their method improved the performance by 6% compared with the unaugmented data. Al Nazi et al. [101] used a VAE model to increase the variations of ECG data. Thiam et al. [23] used deep denoising convolutional autoencoders (DDCAE). Their approach involves optimizing both the joint representation of input channels generated by a multimodal DDCAE and the additional neural network, trained simultaneously, performing the classification task.

Generative Adversarial Networks (GAN): The GAN is a type of DL framework introduced by Ian Goodfellow and his colleagues in June 2014 [137]. GANs became a common way to make new samples be included in the training set. GANs primarily utilize adversarial training to simultaneously optimize two neural networks: a generator and a discriminator. The generator network generates a sample supposed to be similar to those of the original distribution. This is achieved by extracting a random value from a multivariate normal distribution and feed it as input to the generator. The discriminator compares the output from the generator with the original samples and gives out a number between 0 and 1, indicating the probability of identifying a synthetic ECG rather than a real one.

In the context of ECG DA, the authors of [15,17,18,24,94,100,105,107,108,109,110,111,112,114,115,116,117,118,119,122,123,126] used GAN to augment the samples of the minor classes of the MIT-BIH AD. The augmented samples were then fed to a DL model for ECG beat classification, which demonstrated a notable improvement ranging from 0.24–32% compared to the unaugmented samples. While other studies such as [106,113] also employed GANs to augment ECG samples, they used ML-based classifiers, namely, random forest (RF) and support vector machines (SVM), respectively.

Zhou et al. [93] proposed conditional GAN (CGAN) to generate versatile ECG for improving the training efficiency of the DL model. Their methods improved the performance 1.3–2.6% on two different datasets, i.e., MIT-BIH AD and Physionet-2017. Instead of using the ECG signal as a GAN input, some researchers transformed the ECG signal into spectral images. For example, the authors in [11] converted ECG signals into images by using a logarithmic spectrogram.

Xiong et al. [92] designed an ECG generator that consists of three components: clinical ECG recordings, a mathematical model that uses ordinary differential equations, and a 37-layer convolutional recurrent network (CRN) for style transfer. At first, the mathematical model was utilized to create ECG waveforms that represent an idealized heart rate or pacing of the RR intervals using parameters for the mean and standard deviation of the heart rate. These ECG waveforms were then fed into the neural network for style transfer. The authors discovered that their network boosted the accuracy of AF detection by 3% when DA was employed.

Fangyu et al. [102] developed a novel approach to detect abnormal ECG signals with higher accuracy. To address the challenge of imbalanced data affecting model learning, they designed two DA techniques (BiLSTM-CNN and TimeGAN) to improve the semantic information of various features. Additionally, they proposed a contrastive learning framework to ensure consistency in data representation across two different channels. By maximizing the similarity of data representations and calculating contrastive loss, they obtained more complete data category embedding and correlation, which ultimately improved performance by 3% compared to the model without contrastive learning.

Some researchers only used the GANs for ECG synthesis. ECG synthesis has the potential to improve our understanding of the underlying mechanisms of various heart conditions and to develop more accurate diagnostic models. However, it is important to validate the accuracy and reliability of the models built using synthetic ECG signals before deploying them in clinical settings. Based on our search criteria, we found papers that used generative methods only for ECG synthesis; the summary of the methods is tabulated in Table 5. In these papers, different metrics were used to evaluate the performance of GAN models for ECG synthesis. The choice of the metric depends on the specific goals of the research and the characteristics of the generated ECG signals. Some commonly used metrics for evaluating the performance of GAN models for ECG synthesis include:

- Mean Squared Error (MSE) and Root MSE (RMSE): Both MSE and RMSE are based on the average squared difference between the generated ECG signals and the ground truth ECG signals. A lower error indicates better performance.
- Signal-to-Noise Ratio (SNR): The SNR metric calculates the ratio of the signal power to the noise power in the generated ECG signals. A higher SNR value indicates better performance.
- Fré chet Inception Distance (FID): The FID metric measures the distance between the distribution of the generated ECG signals and the distribution of the real ECG signals. A lower FID value indicates better performance.
- Maximum Mean Discrepancy (MMD): The MMD metric measures the distance between two distributions by comparing the mean of their feature representations in a reproducing Kernel Hilbert Space. If the MMD is small, it means that the two distributions are similar in the feature space, and the model trained on one distribution can generalize well to the other distribution.
- Dice Coefficient (DC): The DC metric is used to measure the similarity or overlap between two sets or binary masks. The DC ranges from 0 to 1, where 0 indicates no overlap between the sets and 1 indicates a perfect match.
- Percent Mean Square Difference (PMSD): The PMSD metric is calculated as the square of the difference between the values of the generated and real ECG, divided by the average of the values, and expressed as a percentage. A lower PMSD value indicates better performance.
- Kernel Maximum Mean Difference (KMMD): The KMMD metric is an extension of MMD that maps data to a high-dimensional space using a kernel function to measure similarity between data points. It is used in generative models to evaluate the quality of generated data by comparing them to real data. A high KMMD value means that generated data are different from real data, while a low KMMD value means they are similar.

## 6. Discussion

Small-scale and imbalanced datasets limit the application of AI-based models in cardiology. Undoubtedly, DA is an effective way of solving such problems and has been widely used in various domains. However, DA for ECG signals poses challenges different than in other domains. One of the main problems of applying DA in this context is that ECG signals contain fine-grained information such as relative amplitudes of ECG waveforms (down to a few microvolts) and temporal relationships between data points (down to a few milliseconds). This fine-grained information plays a significant role in the AI-based classifier. Indeed, a synthetic ECG signal can be advantageous for the AI-based model if its fine-grained information is present in the generated sample. Otherwise, DA may have a detrimental effect on the classifier. Universality is another important issue of DA techniques, i.e, DA techniques depend on the input type, input shape (number of data points and number of leads), number of parameters (hyper-parameters) of the AI-based method/DL model, or applications. Moreover, the effectiveness of the same DA varies on ECG rhythms, so applying the same DA techniques for two different types of rhythms could increase performance in one case and degrade performance in another.

Various DA methods have been proposed to generate synthetic ECGs from real ones and improve the performance of the AI-based models. We mostly categorized the DA techniques into two broad categories: basic and advanced. The basic DA techniques are generally simple to apply and relatively fast to compute. Many operations in basic DA showed promising results compared to unaugmented datasets. We however do not recommend the use of time inversion, spatial inversion, permutation, and lead shuffling, whereas a careful design of all other basic DA techniques is needed. For example, scaling the QRS complex of a healthy subject induces symptoms of cardiac hypertrophy. Artificially prolonging the PR interval is instead a symptom of atrioventricular block. In another example, in the context of MI, the lead order is essential for the correct localization of the infarcted area. These techniques may generate non-physiological ECGs or new ECGs belonging to other diagnostic classes, potentially leading to detrimental effects rather than being advantageous. In the field of advanced DA for ECG analysis, researchers have extensively explored the use of GAN-based methods. Of note, most of these works have been on synthesizing or generating ECG beats from the MIT-BIH AD dataset, primarily emphasizing beat-level variations. However, it is important to note that there is limited research available on rhythm generation using advanced DA techniques for specific applications. The focus has primarily been on beat-level augmentation, rather than capturing the broader rhythmic patterns present in ECG data. As a result, it is challenging to generalize which DA techniques would be optimal for specific applications where rhythm generation is the primary concern.

In addition, the improvements obtained vary greatly among different DA techniques, datasets, preprocessing steps, and applications. Generally, it is not possible to determine which augmentation method works best for a given dataset and only empirical tests can drive the selection. On the other hand, advanced DA techniques, such as generative models, can generate higher-quality synthetic data which preserve the statistical properties of the original data distribution, making them more representative and similar to real-world data. Therefore, these advanced DA techniques hold great potential in improving the accuracy and robustness of AI-based models.

In Section 4 and Section 5, we specifically discussed methods to generate ECG signals. However, several studies implemented DL models with spectral images as inputs (see Table 3 and Table 4). With spectral methods, the ECG signals are transformed into spectral images which are then used for classification instead of the raw ECG signals [11,14,26,59,62,95]. DA is performed directly on the spectral images rather than the ECG signals. Despite their use being motivated by the fact that ECG features are often accompanied by changes in frequency band energy, the role of the phase of the sinusoidal components (in which the ECG is decomposed) is overlooked, which could potentially reduce the DL performance. Although spectral methods have shown promising results in certain research settings, interpretability and explainability are further compromised because variations in spectral images cannot straightforwardly be associated with changes in ECG features known to cardiologists.

In our analysis, we also have come to the conclusion that there are still obstacles and challenges that need to be addressed. First, there is currently no clear consensus on the most effective proportion of real and synthetic ECGs to use in order to enhance performance and address overfitting issues. Some studies have investigated the impact of different ratios of real and synthetic ECGs on classification performance and have shown that increasing the number of synthetic ECG samples may not necessarily result in an increase in enhancement effect [46,92,136]. Researchers have used varying ratios of synthetic ECG and real ECG to improve AI-based model performance, but the optimal ratio depends on the specific application and must be determined based on the dataset being used. Therefore, further research is needed to explore the most effective use of real and synthetic ECGs in AI-based models for various applications. Second, quantifying the quality of generated synthetic ECGs is challenging since there is no universal method to quantify its similarity with real ECG signals. How does one quantify whether a synthetic ECG is “real” enough? One method is simply visual inspection: “Does it look right?” However, this often requires domain expertise and cannot be scaled. Most studies have not considered any evaluation scheme to quantify how much closer the synthetic ECG signal is to the real ECG signal (they rely on an increase in classification performance). There are several possible solutions for quantifying the quality of the synthetic ECGs. To begin with, we can extract some important features, i.e., heart rate, the amplitude of the QRS complex, peak-to-peak differences, etc., for every real and synthetic ECG. Then, some distance metrics such as Wasserstein distance, Kullback-–Leibler divergence, or Kolmogorov–-Smirnov could be used to quantify the similarity of the distributions.

In conclusion, we recommend conducting further research to overcome these issues. One promising avenue for exploration is the combination of various approaches of DA in order to expand the datasets. This could involve augmenting data in both the input space and feature space. For instance, adversarial learning can be used to provide secondary augmentation on synthetic ECGs generated using basic DA, potentially leading to a higher degree of variation in the synthetic ECGs. Moreover, combining meta-learning with DA might reveal why DA affects the performance of AI-based model for ECG classification. While DA using adversarial learning is currently popular, it is still important to figure out how to enhance the quality of synthetic ECGs. There is room for development in the areas of improving sample quality and evaluating their efficacy across a variety of datasets.

## 7. Conclusions

Collecting large-scale ECG datasets is challenging due to constraints on available patients, expert cardiologists, recording duration, and operational complexity. Data augmentation can be considered as an effective strategy to augment small-scale datasets and unbalanced minority classes of samples for addressing overfitting and to boost the performance of AI models. The paper discussed the current level of data augmentation research for ECG interpretation using artificial intelligence techniques. After examining the studies, we may conclude that effectiveness of DA methods can vary depending on the specific application. What works well for one application may not be suitable for others. Therefore, advancements in this field necessitate further investigations. In general, we found that data augmentation boosts the performance of automatic ECG analysis. In summary, the paper presented the practical suggestions and performance outcomes presented in the literature. It may provide guidance and help for ECG research and assist to model the inter-patient variability of ECG interpretation.

## Figures and Tables

**Table 1:** List of search queries and the final query.

Parameter	Search Query
Signal type (Q1)	“ECG” OR “electrocardiography” OR “electrocardiogram” OR “EKG”
AI technique (Q2)	“DNN” OR “deep learning” OR “neural network” OR “AI" OR “artificial intelligence” OR “machine learning”
DA technique (Q3)	“augmentation” OR “synthesis” OR “generation”
Specific technique (Q4)	“GAN" OR “generative adversarial network” OR “normalizing flow” OR “stable diffusion”
Final query	Q1 AND Q2 AND (Q3 OR Q4)

**Table 2:** Inclusion and exclusion criteria for selecting papers.

Inclusion Criteria	Exclusion Criteria
Works published in the period between 1 January 2013 and 31 January 2023	Review papers and non-English written papers
Applying DA only to the ECG	Not applying DA and not providing a clear description of DA and datasets
With a clear description of DA	Not considering the ECG signal
Inclusion of AI technique	Not reporting performance metrics

**Table 4:** Summary of advanced DA methods for ECG classification using AI techniques.

Types	Lead	DA Methods	Input	Classifier	Improvem. after DA	Dataset	Refs.
CA	1	Style-transfer	ECG	CRN	3%	Physionet-2017 & Private	[92]
CA	2	CGAN	ECG	CNN	1.3–2.6%	MIT-BIH AD & Physionet-2017	[93]
CA	12	VAE	Spectral	CNN	0–6%	Private	[14]
CA	1	GAN	ECG	CNN	1%	MIT-BIH AD	[94]
CA	1	GAN	ECG	CNN	1.3%	MIT-BIH AD	[15]
CA	1	Embedding space	ECG	CNN	–	Physionet-2017	[48]
CA	1	GAN	Spectral	CBAM-ResNet	–	MIT-BIH AD	[95]
CA	12	Embedding space	ECG	Self-supervised	–	Physionet-2021	[96]
CA	1	GAN	Spectral	CNN	3%	Physionet-2017	[11]
CA	1	GAN	ECG	CNN	–	MIT-BIH AD	[17]
CA	1	GAN	ECG	CNN	5-37%	MIT-BIH AD	[18]
CA	1	GAN	ECG-PPG	CNN	–	BIDMC	[97]
CA	1	MC	ECG	CNN	–	MIT-BIH AD	[12]
CA	1	Embedding space	ECG	CNN	5.8%	ICENTIA11K [98]	[99]
CA	1	GAN	ECG	CNN	–	MIT-BIH AD	[100]
CA	1	VAE	ECG	CNN-LSTM	2%	MIT-BIH AD	[101]
CA	1 & 12	BiLSTM-CNN & TimeGAN	ECG	CNN	–	MIT-BIH AD & PTB	[102]
CA	12	GAN	ECG	ResNet	5%	CPSC-2018	[103]
CA	1	GAN	ECG	CRNN	14%	Physionet-2017	[104]
CA	1	GAN	ECG	Bi-LSTM	1.9%	MIT-BIH AD	[105]
CA	1	GAN	ECG	RF	11%	MIT-BIH AD	[106]
CA	1	GAN	ECG	LSTM	–	MIT-BIH AD & MIT-BIH NSR	[107]
CA	1	GAN	ECG	CNN	1.45%	MIT-BIH AD	[108]
CA	1	GAN	ECG	CNN	–	MIT-BIH AD	[109]
CA	1	GAN	ECG	CNN-LSTM	2.65%	MIT-BIH AD	[110]
CA	1 & 12	GAN	ECG	CNN	–	MIT-BIH AD & PTB	[111]
CA	1	GAN	ECG	CNN	0.24%	MIT-BIH AD	[112]
CA	2	GAN	ECG	SVM	32%	MIT-BIH AD	[113]
CA	1	GAN	ECG	Bi-LSTM	2–51%	MIT-BIH AD	[114]
CA	1	VAE & GAN	ECG	CNN	5%	MIT-BIH AD	[115]
CA	1	GAN	ECG	CNN	–	MIT-BIH AD	[116]
CA	1	GAN	ECG	CNN	–	MIT-BIH AD	[117]
CA	1	GAN	ECG	LSTM	–	MIT-BIH AD	[118]
CA	1	GAN	ECG	ResNet-BiLSTM-attention	–	MIT-BIH AD	[119]
CA	1	AE	ECG	CNN	–	Physionet-2017	[120]
CA	1	GAN	Spectral	CNN	–	MIT-BIH AD	[121]
CA	1	GAN	ECG	Multi-head Attention	5–10%	MIT-BIH AD	[122]
CA	1	GAN	ECG	CNN	–	MIT-BIH AD	[123]
CA	1	GAN	ECG	CNN	32%	MIT-BIH AD	[113]
CA	1	BiRNN	ECG	Ensemble Bagged Trees	–	MIT-BIH AD	[124]
CA	1	GAN	ECG	CNN	4.8–8.1%	Private	[125]
CA	1	GAN	ECG	LSTM	4%	MIT-BIH AD	[126]
CA	1	GMM	ECG	ResNet	6.7%	MIT-BIH AD	[127]
CA	12	Embedding space	Spectral	Self-supervised	–	Private	[128]
CA	1	GAN	ECG	CNN	–	AHADB, VFDB, & CUDB	[129]
MI	1	Encoder-decoder	ECG	CNN	–	PTB	[130]
MI	12	Wasserstein Geodesic Perturbation	ECG	MFT	6–17%	PTB-XL	[13]
MI	1	GAN	ECG	CNN	4–6%	PTB	[131]
Fetal	1	GAN	ECG	CNN	12%	CTU-UHB	[132]
Emotion	1	GAN	ECG	LSTM	17%	CASE	[133]
Biometric	1	GAN	ECG	CNN	–	ECG-ID	[134]
Sleep-Apnea	1	GAN	ECG	CNN-LSTM	1.78	Apnea-ECG & MIT-BIH AD	[24]
Emotion	–	GAN	ECG	CNN	5.64%	Private	[135]
MI	12	GAN	ECG	SVM	0.75%	PTB	[136]
Emotion	1	GAN	ECG	SVM	–	DECAF	[22]
Pain intensity	1	DDCAE	ECG	NN	–	BioVid Heat Pain	[23]

**Table 5:** Summary of generative methods for ECG synthesis using AI techniques.

Lead	Input	Method	Metric	Dataset	Refs.
1	ECG	GAN	MMD (3.83 $\times \;10^{-3}$)	LUDB [138]	[139]
1	ECG	GAN	KMMD (5.53)	MIT-BIH AD	[140]
1	ECG	GAN	MSE (0.017–0.099)	PTB-XL	[141]
1	ECG	GAN	SNR (40.85 dB)	MIT-BIH AD	[142]
1	ECG	GAN	RMSE (0.126)	MIT-BIH AD	[143]
1	ECG	AE	MSE (0.2)	MIT-BIH AD	[144]
1	ECG	GAN	FID (4.77–17.19)	MIT-BIH AD	[145]
2	ECG	GAN	PMSD (7.21%)	–	[146]
1	ECG	BiLSTM-CNN GAN	RMSE (0.276)	–	[147]
12	ECG	U-Net generator	DC (0.868)	Private and INCART	[148]
1	ECG	GAN	RMSE (0.015–0.028)	MIT-BIH AD	[149]
12	ECG	Genetic Algorithm-NN	RMSE (44.9–90) μV	PTB	[150]
12	ECG	CycleGAN	MSE ([0.5–31] $\times 10^{-3}$)	Private	[151]

## Data Availability

The data generated and analyzed during this study are available from the corresponding author by request.
